# Mitochondrial DNA and Inflammation in Alzheimer’s Disease

**DOI:** 10.3390/cimb45110540

**Published:** 2023-10-25

**Authors:** Giacoma Galizzi, Marta Di Carlo

**Affiliations:** Institute for Research and Biomedical Innovation (IRIB), National Research Council (CNR), Via Ugo La Malfa, 153-90146 Palermo, Italy; marta.dicarlo@cnr.it

**Keywords:** mitochondria, mitochondrial dysfunction, neuroinflammation, neurodegeneration, glia, microglia, mtDNA, DAMPs, Alzheimer’s disease

## Abstract

Mitochondrial dysfunction and neuroinflammation are implicated in the pathogenesis of most neurodegenerative diseases, such as Alzheimer’s disease (AD). In fact, although a growing number of studies show crosstalk between these two processes, there remain numerous gaps in our knowledge of the mechanisms involved, which requires further clarification. On the one hand, mitochondrial dysfunction may lead to the release of mitochondrial damage-associated molecular patterns (mtDAMPs) which are recognized by microglial immune receptors and contribute to neuroinflammation progression. On the other hand, inflammatory molecules released by glial cells can influence and regulate mitochondrial function. A deeper understanding of these mechanisms may help identify biomarkers and molecular targets useful for the treatment of neurodegenerative diseases. This review of works published in recent years is focused on the description of the mitochondrial contribution to neuroinflammation and neurodegeneration, with particular attention to mitochondrial DNA (mtDNA) and AD.

## 1. Introduction

Alzheimer’s disease (AD) is characterized by the accumulation of amyloid-β (Aβ) plaques and hyperphosphorylated tau neurofibrillary tangles (NFTs) in the brain, resulting in progressive neuronal death and synaptic dysfunction. Neuroinflammation and mitochondrial dysfunction, which includes mitochondrial damage and dysfunctional mitophagy, are currently considered critical components in the pathogenesis of AD [1,2,3].

In recent years, there has been increasing talk about “mitoinflammation”, an inflammatory response mediated by mitochondria that seems to play an important role in the pathogenesis of various neurodegenerative diseases, including AD.

In addition to stimulating neuroinflammation [4,5], the accumulation of toxic protein aggregates, such as Aβ and NFTs, induces mitochondrial damage, which creates leakage of mitochondrial DNA (mtDNA) into the cytoplasm [6], promoting an inflammatory response [7].

Neuroinflammation is a defensive response of the brain to harmful stimuli of various origins, such as infections, trauma, protein aggregation and accumulation [8].

In the beginning, the neuroinflammatory response exerts a protective effect due to its ability to remove cellular debris and promote tissue regeneration. In the long term, instead, the persistence of a chronic neuroinflammatory state, with prolonged release of pro-inflammatory mediators, can cause synaptic dysfunction and neuronal death. Thus, neuroinflammation can participate in the development of neurodegenerative diseases [9]. In light of numerous studies, neuroinflammation is therefore considered a common element in various neurodegenerative pathologies, both acute and chronic (e.g., stroke, vasculitis, AD, Parkinson’s disease (PD), amyotrophic lateral sclerosis (ALS), multiple sclerosis), including aging. Therefore, it is not surprising that, in recent decades, an increasing number of studies on neuroinflammation have been carried out with the aim of creating the basis for future broad-spectrum therapeutic approaches [10].

The main players in neuroinflammatory processes are glial cells, which are classified as macroglia (oligodendrocytes and astrocytes) and microglia. They, activated by the presence of pathogens or extraneous substances, are responsible for the innate and adaptive immune responses [11]. Since mitochondria are organelles of symbiotic origin, they are able to trigger, through the release of mitochondrial damage-associated molecular patterns (mtDAMPs), an innate immune response [8].

These mtDAMPs are mitochondrial components, including mtDNA, mitochondrial transcription factor A (TFAM), ATP, cytochrome c, and cardiolipin, released into the cytoplasm and extracellular environment when severely damaged mitochondria are not correctly removed by mitophagy. mtDAMPs are recognized by microglial receptors and can induce an immune response with the release of pro-inflammatory cytokines [10].

This review, in addition to a brief description of neuroinflammation, provides an overview on the mitochondrial contribution to neuroinflammation by reporting the inflammatory pathways linked to mitochondrial damage, with a focus on the role of mtDNA in the neuroinflammatory response in AD.

## 2. The Role of Microglia in Neurodegeneration

Neuroinflammation is a cellular defense mechanism that is triggered as a result of the loss of brain homeostasis. It involves an initial pro-inflammatory phase, which attempts to neutralize the insult, followed by an anti-inflammatory phase. This phase, through the activation of regenerative mechanisms, tends to restore the correct functional structure of the neuronal tissue, repairing the damage and restoring synaptic functionality. When the harmful stimulus persists, as happens in neurodegenerative diseases, it moves towards chronic neuroinflammation with an enhancement of the pro-inflammatory phase that overcomes the regenerative phase, leading to tissue damage [11]. This supports the idea of a close relationship between neuroinflammation and neurodegeneration events, and suggests an early activation of neuroinflammation that could precede and initiate neuronal degeneration [12,13].

Neuroinflammation is a well-coordinated event in which microglia cooperate with other cells present in the nervous tissue, such as astrocytes, capillary endothelial cells, and infiltrating blood cells that arise when the blood–brain barrier (BBB) is no longer efficient, as often happens in neurodegenerative disorders and aging [14].

Neuroinflammation is regulated by specific brain immune and inflammatory cells specialized in counteracting pathogens and/or tissue insults thanks to the presence of different pattern recognition receptors (PRRs). These cells, mainly microglia and astrocytes, represent respectively about 5–15% and 20% of the total central nervous system (CNS) cells [15].

Microglia are considered the resident macrophages of the CNS. The morphology of microglia varies depending on their active or inactive state. In fact, they exhibit a branched conformation with numerous thin and elongated cytoplasmic processes that allow them to carry out immune surveillance in the surrounding environment and interact with nearby cells, which is typical of the resting form. Activated microglia instead present thickened processes and an increasingly reduced degree of branching, until they reach an amoeboid morphology (without branches and with a rounded soma) with great phagocytic capacity [14,16]. At the molecular level, the quiescent state in microglia is characterized by a low expression of cluster of differentiation 68 (CD68) and major histocompatibility complex I and II (MHC-I, MHC-II). In contrast, the activated microglia express high levels of MHC-II and co-stimulatory antigens that have the function of presenting antigens to naïve T cells and triggering pathways that regulate the production of inflammatory molecules [16].

From a study by Vela et al. on the distribution and morphology of microglial cells in the cerebellum of wild-type mice of different ages, it can be seen that microglia can present different phenotypes, each linked to specific functional properties [17]. Thus, to highlight its dichotomous activity, in some reports we find microglia simplistically classified as M1 pro-inflammatory phenotype and M2 anti-inflammatory phenotype [18,19], which in turn is further divided into four subtypes, M2a, M2b, M2c and M2d [20].

M1 microglia are mainly activated in response to high levels of interferon-γ (IFN-γ) and tumor necrosis factor-α (TNF-α), produced by natural killer cells and T-helper 1 lymphocytes. They are characterized by several cell surface markers (such as CD16, CD32, CD40 and CD86) and show their pro-inflammatory response by expressing the interleukins IL-1β, IL-6, IL-8 and TNF-α [21], oxygen and nitrogen free radicals, and promoting microglial phagocytosis of damaged cells and of neurotoxic aggregates [21,22]. M2 microglia are characterized by cell surface markers such as CD206, CD163, and arginase. It induces anti-inflammatory responses by expressing IL-10, IL-4, IL-13, and transforming growth factor-β (TGF-β) [23,24], which can inhibit phagocytosis inflammation, as well as induce tissue repair.

The activation of glial cells occurs in response to stimuli of different natures, such as microbial products, cytokines, products released by damaged neurons and disease-related proteins (e.g., Aβ, tau/p-tau or α-synuclein) [25].

As mentioned previously, activated glial cells have a dichotomous action; in fact, in acute contexts, they can be protective, eliminating debris and releasing neurotrophic factors that contribute to the activation of tissue regeneration mechanisms. However, excessive activation of glial cells, which occurs during neurodegenerative diseases, can, through the release of potentially neurotoxic mediators including cytokines (TNF-α, IL-6, IL-1β, IL1-α), chemokines (RANTES and MCP-1), reactive oxygen species (ROS), nitric oxide (NO), proteolytic enzymes and glutamate, feed a neuroinflammatory state by damaging adjacent neurons [26].

In fact, to maintain CNS homeostasis, continuous communication between microglia and surrounding cells, such as astrocytes and neurons, is necessary [27]. Therefore, in astrocytes, inflammatory cytokines such as IL-1α, IL-1β, and TNF-α, secreted by active microglia, can induce an inflammatory response [28,29].

Astrocytes are the most numerous glial cells in the CNS, where they perform various functions ranging from the regulation of synaptic plasticity to the maintenance of the BBB [30,31]. Similarly to microglia, they can present a pro-inflammatory phenotype (A1), which induces inflammatory factors such as IL-1β, TNF-α and NO, and a neuroprotective phenotype (A2) capable of releasing protective and neurotrophic factors, such as IL-4, IL-10, active TGF-β1 and brain-derived neurotrophic factor (BDNF), to improve cell survival [1,29,32].

The activation of glial cells occurs through PRRs capable of activating various signaling pathways that mediate the inflammatory process and phagocytosis [15]. PRRs belong to families of membrane-bound receptors, which include Toll-like receptors (TLRs), nucleotide-binding oligomerization domain (NOD)-like receptors (NLRs), C-type lectin receptors (CLRs), Retinoic acid-inducible gene I (RIG-I)-like receptors (RLRs), absent in melanoma-2 (AIM-2)-like receptors (ALRs), formyl peptide receptors, and scavenger receptors [33].

Activated microglia initiate several signaling pathways, such as phosphoinositide 3-kinase/protein kinase B (PI3K/AKT), mitogen-activated protein kinase (MAPK), and mammalian target of rapamycin (mTOR) that leads, among other things, to nuclear factor κB (NF-κB) activation. NF-κB translocates to the nucleus where it initiates the production of pro-inflammatory cytokines and chemokines, inducible enzymes that feed neuroinflammation [34,35].

There is a growing body of literature suggesting the involvement of glia in the pathogenesis of AD. Microglia and astrocytes interact with Aβ oligomers and fibrils, modifying their conformation and phenotype. The presence of non-functional microglia can cause an accumulation of Aβ [29]. For example, genetic mutations in the triggering receptor expressed on myeloid cells 2 (TREM2) increase the risk of developing late-onset AD (LOAD). TREM2 is present on microglial membranes, and it is involved in the phagocytosis of microglial cells. The mutation found in AD and the reduction of TREM2 activity decreases the autophagocytic capacity of microglia, causing an increase in amyloid plaques [36,37,38].

In recent years, several studies have confirmed the presence of high levels of various cytokines, such as IL-1β, IL-6, IL-18, IL-33 and TNF-α, in various neurodegenerative disorders, including AD [39]. The continuous production of these inflammatory molecules culminates in neuronal death and synaptic dysfunction. For example, it has been shown that the activation of NF-κB induces an upregulation of amyloid precursor protein (APP) in neurons and increased production of Aβ, confirming the pathogenetic role of neuroinflammation in AD [40].

The dual role of microglial activation in the pathogenesis of AD is increasingly recognized. On the one hand, microglia contribute to the production of Aβ and the formation of amyloid plaques through the release of pro-inflammatory mediators. On the other hand, microglia play a neuroprotective role by activating the removal of Aβ plaques and the production of neurotrophic factors [41].

## 3. Mitochondria, between Neurodegeneration and Neuroinflammation

The fundamental role of mitochondria for cellular homeostasis has been widely described and documented in recent years. Their involvement in a large variety of cellular functions is now known. In fact, they regulate the production of ATP through oxidative phosphorylation, the metabolism, the homeostasis of intracellular calcium, the production of ROS, the synthesis of steroids, the catabolism of fatty acids, cell proliferation and apoptosis [42,43].

Neurons are particularly vulnerable to mitochondrial damage because their ability to produce neurotransmitters and maintain membrane excitability depends on them. Indeed, mitochondria are fundamental in the regulation of presynaptic calcium in central glutamatergic terminals [44].

Therefore, the accumulation of dysfunctional mitochondria leads to cell damage and neuron degeneration. Furthermore, in recent years, the ability of mitochondria to react to cellular damage has emerged, promoting, thanks to their endosymbiotic nature, the host’s immune response. Thus, it is not surprising that more and more studies have supported the central role of these organelles in the pathogenesis of CNS disorders also through direct action in neuroinflammation. Several studies have shown an early involvement of mitochondrial damage in cellular and animal models of AD, with a decrease in mitochondrial respiration and a reduction on the activity of complex I, complex II–III, and cytochrome oxidase preceding the accumulation of Aβ in AD mice [45,46,47,48]. Furthermore, alterations in mitochondrial number, morphology, and activity have been highlighted, culminating in an aberrant production of mitochondrial ROS (mtROS) [7,49]. For example, analyzing the frontal cortex of patients with early, definite, and severe AD, Manczak et al. found an imbalance in mitochondrial dynamics with overexpression of the mitochondrial fission gene and downregulation of the fusion gene, likely due to the interaction between dynamin-related protein 1 (DRP1) and Aβ, suggesting a role of mitochondria in neuronal health and synaptic damage [50].

mtROS, produced by the electron transport chain during ATP production, is considered an important cell-signaling molecule. The overproduction of mtROS is responsible for oxidative damage to proteins, lipids, and nucleic acids, including mtDNA. In this way, it drives mitochondrial dysfunction and apoptosis, and contributes to the progression of several neurodegenerative diseases [47,51]. Furthermore, oxidative stress is responsible for the fragmentation and release of mtDNA from mitochondria to the cytosol and extracellular space, where it acts as a potent inducer of inflammation [7,52,53].

Damaged mitochondria undergo mitochondrial quality control, a mechanism involved in the isolation and destruction of dysfunctional mitochondria via selective autophagy known as mitophagy. We have mentioned this mechanism in detail in a previous review [54]. Neurodegenerative diseases, such as AD and PD, are often associated with an alteration of the mitophagic process and the intracellular accumulation of damaged mitochondria [3]. Dysfunctional mitochondria lose mitochondrial membrane integrity and release mtDAMPs into the cytoplasm and extracellular space [8,55,56]. Among the mtDAMPs molecules of different nature, such as mtDNA, cardiolipin, cytochrome c (CytC), TFAM, and N-formyl peptides, are present. Several studies report the involvement of DAMPs in the neuroinflammatory process. They directly stimulate the PRRs present on microglia and astrocytes [57], triggering an innate immune mechanism that contributes to neuron degeneration [57,58,59].

In the last few years, an emerging role of mitochondria in the pathogenesis of neurodegenerative diseases concerning their neuroinflammatory capacity has been increasingly recognized. Therefore, a deeper understanding of the mechanisms underlying the ability of mtDAMPs to regulate the complex neuroinflammatory machinery can make an important contribution to the identification of new therapeutic targets for the treatment of AD.

## 4. mtDNA, a Mitochondrial DAMP with Great Potential

Mitochondria are intracellular organelles of endosymbiotic origin and possess bacterial characteristics such as the presence of lipid cardiolipin in the membrane, N-formylated peptides, and double-stranded circular DNA with a hypomethylated cytosine–phosphate–guanine (CpG) motif [8,60,61].

Each mitochondrion contains a large number of mtDNA copies per cell. Being close to the electron transport chain, mtDNA easily undergoes oxidation and therefore has a propensity towards mutations [62].

Events such as the accumulation of mtDNA mutations, increased ROS levels, imbalances in mitochondrial dynamics, and loss of mitochondrial membrane potential exacerbate mitochondrial dysfunction and lead to the release of mtDNA. Defective mitochondria are usually degraded by mitophagy, but if stress persists, damaged mitochondria can escape this quality-control pathway and undergo structural modifications that allow the leakage of mitochondrial components. mtDNA released from distressed neurons can act on astrocytes and microglia to induce neuroinflammation [63]. Therefore, autophagy/mitophagy may represent a control mechanism for the inflammatory process.

**Figure 1:**
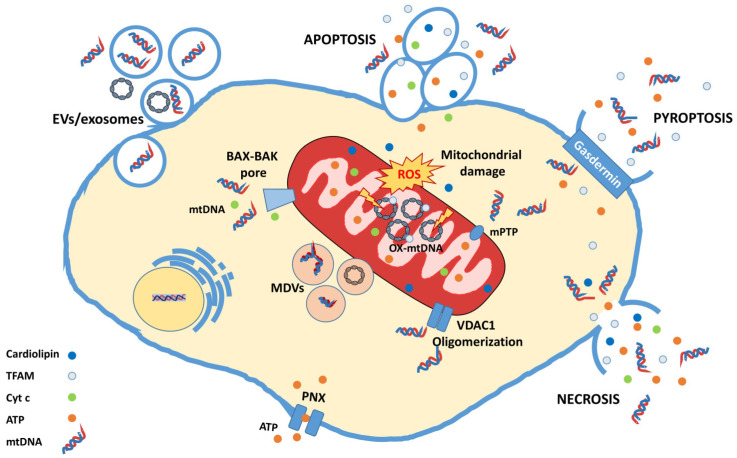
**Mitochondrial DAMPs release mechanisms.** The figure shows a schematic representation of mtDAMPs release mechanisms. mtDAMPs can be released by mitochondria into the cytosol (mPTP, BAK-BAX pore, VDAC1 oligomerization) or in the extracellular space by passive (apoptosis, pyroptosis and necrosis) and active release mechanisms (extracellular vesicles (EVs), exosomes, mitochondria-derived vesicles (MDVs), and pannexin channel (PNX)).

Several studies have attempted to explore how mtDNA is released outside the mitochondrion (Figure 1). Garcia et al. conducted studies on rat liver cells exposed to oxidative stress, and observed mtDNA release mediated by the opening of the mitochondrial permeability transition pore (mPTP) [64]. Similarly, McArthur et al., using light sheet microscopy, observed in mouse embryonic fibroblasts that, following activation, BAK and BAX oligomerize, forming large pores on the outer mitochondrial membrane from which components of the mitochondrial matrix, including mtDNA, leak out [65].

Kim et al. proposed that the release of mtDNA from mitochondria subjected to oxidative stress can also occur through the formation of pores on the outer mitochondrial membrane by voltage-dependent anion channel 1 (VDAC1) oligomerization [53].

In addition to being freed molecules, mtDNA can also be released inside vesicles of various kinds, including mitochondria-derived vesicles (MDV) or exosomes [10,66]. These vesicles are able to activate the inflammatory response in immune cells through the cGAS/STING pathway [67].

### 4.1. Neuroinflammation Activated by mtDNA

Sterile inflammation occurs in the absence of pathogens and begins when PRRs bind to DAMPs released following cellular damage. PRRs are expressed on different cell types involved in the inflammatory response, such as macrophages, neutrophils, dendritic cells, microglia, and astrocytes [7].

mtDNA present in the extracellular environment is recognized as a DAMP by glial cells, triggering an immune response through the activation of several PRRs, such as cyclic guanosine monophosphate-adenosine monophosphate (GMP-AMP) synthase (cGAS), and the NLRP3 inflammasome, present in the intracellular compartments, and TLRs expressed on the cell membrane [8,68,69,70].

#### 4.1.1. cGAS-STING

cGAS is a cytosolic double-stranded DNA (dsDNA) sensor, predominantly expressed in microglial cells [6]. It is capable of triggering the type I interferon (IFN-I) pathway, culminating with the induction of the expression of IFN-I (which includes IFN-α and -β) and inflammatory cytokines by the translocation of interferon regulatory factor 3 (IRF3) and NF-ĸB to the nucleus [71]. Since mtDNA has a double-stranded structure, it is a central activator of cGAS-STING signaling [72].

**Figure 2:**
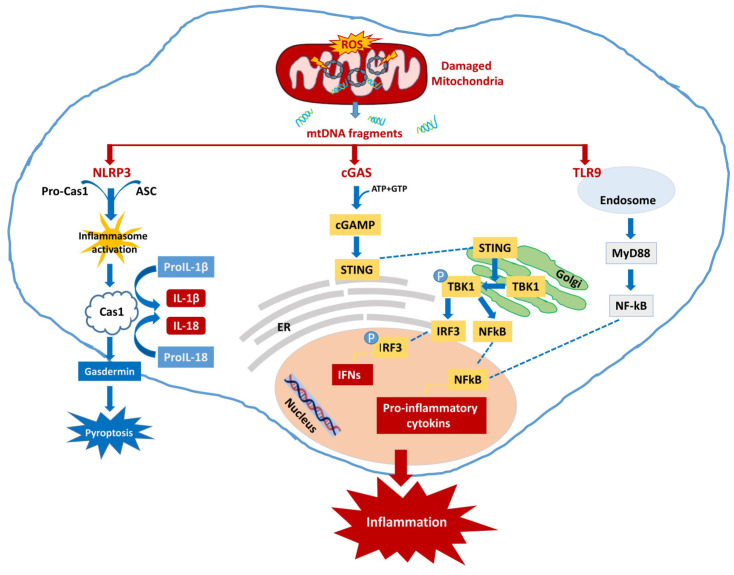
**Inflammatory pathways driven by mtDNA.** Following cellular stress, mitochondria are damaged leading to the accumulation and release of oxidized mtDNA fragments. mtDNA triggers an inflammatory response via cytosolic NLRP3 or cGAS-STING pathways, or via endosomal localized TLR9 signaling. In turn, it promotes the activation of NF-kB and the transcription of pro-inflammatory genes, such as interferons (IFNs), and pro-inflammatory interleukins.

Briefly, cGAS is constitutively present as an inactive protein mainly in microglial cells. Contact with dsDNA induces a conformational change in the cGAS protein which allows interaction with ATP and GTP, and the production of the second messenger cyclic GMP-AMP (cGAMP). cGAMP activates the stimulator of interferon genes (STING) protein which translocates from the endoplasmic reticulum (ER) to the Golgi compartment where it binds protein kinase 1 (TBK1), promoting its autophosphorylation [6,73,74]. The STING-TBK1 complex, through the translocation of the IRF3 transcription factor and NF-ĸB to the nucleus, promotes the production of IFN-I and the genes encoding inflammatory cytokines, including IL-6 and TNF-α [75,76] (Figure 2).

Several studies have shown the involvement of the cGAS-STING pathway in AD, suggesting a role in its onset and progression [77]. Studies conducted by Xie et al. in the 5xFAD mouse model of AD showed colocalization between phosphorylated STING and the activated microglial marker CD68 around Aβ plaques, and found increased interactions between cGAS and dsDNA in both the human AD brain and the 5xFAD mouse [78]. Additionally, treatment with H-151, a STING inhibitor, reduced inflammation and the presence of Aβ_42_ in the cortex of 5xFAD mice. H-151 also decreased Aβ_42_-induced IL-6 production in human HMC3 microglial cells [78]. Furthermore, increased phosphorylation of STING, TBK1, p-65, and IRF3 was measured in the prefrontal cortex of a patient with AD. Moreover, elevated levels of IFN-I have been measured in human AD brains postmortem [6].

Similarly, Hou et al. showed increased levels of cGAS and STING protein expression in the brains of APP/PS1 mice compared to wild-type. In contrast, genetic deletion of the cGAS gene improved neuroinflammation and reduced cognitive impairment [79].

Another study conducted in 5xFAD mice confirmed an upregulation of the cGAS-STING pathway in an AD model. The silencing of microglial cGAS in the early phase of the pathology significantly limited plaque formation, preserved synaptic integrity, and protected mice from Aβ-induced cognitive impairment. Thus, it suggests that cGAS-STING signaling may have an important role in activating the pro-inflammatory microglial phenotype that drives the pathology [80].

Post-mortem human AD brain samples showed increased expression of STING in neurons adjacent to amyloid plaques compared to age-matched control brain samples [6]. Furthermore, IFN signaling is increased in AD brains, and the analysis of healthy and AD postmortem brain samples highlighted the upregulation of phosphorylated TBK1 levels in AD brains [80,81,82].

Similarly, a study of AD conducted on different mouse models at different ages found an upregulation of IFN-I response genes, together with early memory decline and a progressive accumulation of Aβ [80,83], while the genetic ablation of *Cgas* in a mouse model of tauopathy reduced the microglial IFN-I response, preserved synapse integrity, and improved cognitive impairment [84].

IFN-I binds to the interferon alpha receptor (IFNAR), composed of the transmembrane subunits IFNAR1 and IFNAR2, to activate the proteins Janus kinase (JAK), tyrosine kinase 2 (TYK2), and signal transducer and activator of transcription (STAT). Once phosphorylated, STAT move to the nucleus further regulating immune cell recruitment and inflammatory progression. It is therefore possible to reduce neuroinflammation by acting on this pathway. In fact, various studies have reported that the downregulation of IFNAR1 leads to an improvement in astrocytic activity, a decrease in IFN-I and pro-inflammatory cytokines, and an attenuation of microglial proliferation around amyloid plaques [85]. Similarly, the ablation of IFNAR1 and IRF7 in the APP/PS1 transgenic mouse provides some protection from Aβ-induced neurotoxicity [86,87].

Finally, the ability of molecules involved in the cGAS-STING pathway to interact with beclin-1 for promoting mitophagy in innate immune cells and increase mtDNA degradation is interesting [88]. In Parkin or Pink1 knockout mice, an inflammatory phenotype that can be alleviated by genetic inactivation of STING has been established [89]. These findings suggest that the cGAS-STING pathway can be a potent therapeutic target to control mitoinflammation through mitophagy.

These data confirm the importance of the cGAS-STING pathway in neuroinflammation related to AD pathology. It is therefore worth pursuing further studies in this field to find therapeutic targets capable of improving the pathological condition of AD.

#### 4.1.2. NLRP3

The nod-like receptor (NLR) family represents another DAMP sensor belonging to PRRs. Some NLRs, once activated by interacting with DAMPs, form a multiprotein complex called an “inflammasome”. In general, an inflammasome consists of a molecular receptor NLR, an adapter protein, and the caspase-1 precursor. Inflammasomes allow the activation of caspase-1 and the subsequent maturation and release of the pro-inflammatory cytokines IL-1β and IL-18 [90,91].

Inflammasome activation requires two stimuli: a priming signal provided by an inflammatory stimulus, such as TLRs and the TNF-α receptor, leading to NF-κB-mediated NLRP3 expression and the upregulation of pro-IL-1β and pro-IL18. An activation or danger signal, provided by pathogen-associated molecular patterns (PAMPs) or DAMPs, promote inflammasome assembly [92,93].

NLRP3, the most studied inflammasome, is mainly present in microglial cells [94]. When inactive, NLRP3 localizes to the ER membrane and the cytosol, but when both NLRP3 and its adapter ASC (apoptosis-associated speck-like protein containing a caspase recruitment domain (CARD)), are activated, they are relocated to the mitochondria-associated membrane (MAM) fraction. Here, they can detect ROS and DAMPs produced by damaged mitochondria, such as mtDNA [95].

The NLRP3 inflammasome consists of the NLRP3, ASC, and caspase-1 precursor proteins. Upon inflammasome activation, caspase-1 converts pro-IL-1β, pro-IL-18 and gasdermin-D (GSDMD), a pyroptosis inducer, into their active forms [96]. NLRP3 is a multimeric protein consisting of a conserved core nucleotide-binding and oligomerization domain (NOD or NACHT), a C-terminal leucine-rich repeat (LRR) domain, and an N-terminal pyrin domain (PYD). The NOD domain, thanks to its ATPase activity, is necessary for the self-oligomerization of the molecules at the beginning of the inflammasome assembly. The LRR domain is essential for recognizing PAMPs and DAMPs, and maintaining the NLRP inactive state [90,91]. ASC recruits pro-caspase-1 through CARD-CARD interaction. When pro-caspase-1 molecules come together, they undergo an autocatalytic cleavage process that cuts pro-caspase-1 into the p20 and p10 subunits. These subunits bind another identical set of subunits to form an active tetramer. Once activated, caspase-1 cleaves pro-IL-1β and pro-IL-18 into their active forms (IL-1β and IL-18] and induces their secretion [96,97].

The release of the cytokines IL-1β and IL-18 induces the activation of the pro-inflammatory microglial M1 phenotype [11,93]. Furthermore, the NLRP3 inflammasome, through the production of GSDMD, triggers a form of pro-inflammatory cell death known as pyroptosis [96] (Figure 2). Gasdermines can bind to membrane lipids, altering their integrity, creating pores in the cell membrane, and facilitating the secretion of the inflammatory cytokines IL-1β and IL-18, and many intracellular DAMPs that can trigger the inflammatory process in nearby cells, increasing any ongoing neuroinflammation with a feed-forward mechanism [98,99,100].

Studies conducted in recent years have shown the involvement of mitochondrial damage in the activation of the NLRP3 inflammasome, identifying mtDNA as the main activator [100,101,102]. It has recently been observed, in cellular and mouse models of AD and in the brains of human patients, that the amyloid-beta peptide is also able to activate microglial NLRP3 inflammasomes [103,104].

The activation of the NLRP3 inflammasome is involved in the pathogenesis of various neurodegenerative diseases, including AD [103,105]. The use of NLRP3 and caspase-1 knockout mice demonstrated the involvement of the NLRP3/caspase-1 axis in the pathogenesis of AD. Indeed, APP/PS1 mice deficient in caspase-1 or NLRP3 showed a significant improvement in spatial memory and hippocampal synaptic plasticity [106,107,108]. Inversely, increased expression of caspase-1 and NLRP3 genes promotes Aβ accumulation and facilitates lesion production in the brains of APP/PS-1 transgenic mice [109].

Furthermore, high levels of IL-1β have been found in the serum, cerebrospinal fluid, and brains of patients with AD and other types of dementia [110,111,112,113,114]. Once released, the effector molecule IL-1β increases the production of Aβ by neurons and participates in the phosphorylation of the tau protein [115]. Consistently, the inhibition of IL-1β release reduced neuroinflammation and the accumulation of Aβ and tau, and improved cognitive dysfunction and memory in 3xTg-AD mice [116].

Similarly, high levels of IL-18, the other pro-inflammatory cytokine released following activation of the NLRP3 inflammasome, have been found in the bodily fluids of patients with mild cognitive decline and AD [113,114,117]. Furthermore, the involvement of IL-18 in tau hyperphosphorylation through glycogen synthase kinase 3β (GSK-3β) and cyclin kinase 5 has been demonstrated [118].

It seems that the activation of the NLRP3 inflammasome occurs already in the early stages of AD pathology. Indeed, patients with early or mild stages of AD showed higher levels of IL-1β and caspase-1 compared to age-matched controls [119,120].

An interesting study supporting the role of microglia in the clearance of Aβ plaques reports that the inflammasome components NLRP3 and caspase-1 colocalize with p-Tau and Aβ in glial cells, as well as the produced cytokines IL-1β and IL-18, which are more highly expressed in the temporal cortex of the post-mortem AD brain [114,121]. Furthermore, cytokines produced by the activation of the microglial NLRP3 inflammasome are able to induce the inflammatory response of astrocytes responsible for neuronal damage and synaptic dysfunction in AD models [28,122].

Therefore, based on the numerous findings that show the inflammasome to be associated with a neuroinflammatory response and the pathogenesis of AD, attention has been focused on the NLRP3 inflammasome as a possible therapeutic target for treatment of AD. Several inhibitors have been tested in vivo with encouraging results. For example, MCC950 (also known as CRID3 and CP-456773) is an experimental drug capable of inhibiting NLRP3. It has been shown to attenuate the activation of reactive microglia in a mouse model of sporadic AD caused by streptozotocin [123] and to improve cognitive function in APP/PS1 and SAMP8 mouse models of AD [124,125]. Recently, MCC950 and other NLRP3 inhibitors, such as Inzomelid, have undergone phase 1 clinical trials for AD with encouraging results [126]. JC124, another small molecule inhibitor of the NLRP3 inflammasome, works by blocking the caspase-1 activation and secretion of IL-1β. The inhibition of the NLRP3 inflammasome with JC124 in APP/PS1 mice significantly reduced Aβ plaques and neuroinflammation in APP/PS1 mice, leading to improved synaptic plasticity and cognitive function [127].

VX-765, also known as Belnacasan, is a BBB-permeable caspase-1 inhibitor that has already been approved by the Food and Drug Administration (FDA) for clinical trials in humans. This molecule, tested on a J20 a mouse model overexpressing human APP with a mutation linked to familiar AD, and on Sprague–Dawley rats, improved memory capacity, blocked Aβ deposition, improved neuroinflammation, and re-established synaptophysin levels in the mouse hippocampus [128,129].

#### 4.1.3. TLR

Astrocytes and microglia may also be activated via another family of PRRs, called TLRs, which are found expressed in different immune cell populations, including B cells, dendritic cells, and cells of the monocyte/macrophage lineage, such as microglial cells, where they localize in the endosomal vesicles [130]. Their expression varies among immune cells. In human AD brain immune cells, TLR mRNA is overexpressed compared to the healthy brain, with the exception of TLR2, which remains unchanged [131]. In humans, the TLR family (TLR1 to TLR10) is mainly composed of type I transmembrane glycoproteins and can be located both on the cell surface, like TLR1, 2, 4, 5, 6 and 10, and in membrane intracellular cells, like TLR 3, 7, 8 and 9 [132,133]. Each one detects distinct external pathogen-associated molecular patterns or internal damage-associated molecular patterns. For example, bacterial lipopolysaccharide (LPS) is recognized by TLR4, lipoproteins by TLR2, flagellin by TLR5, single-stranded viral RNA (ssRNA) by TLR7, and double-stranded viral RNA (dsRNA) by TLR3 [130,134].

TLR9 recognizes the hypomethylated CpG motif typical of bacterial DNA and mtDNA [8]. mtDNA contains an unmethylated or hypomethylated CpG sequence. These sequences are TLR9 ligands that are recognized by TLR9 in the endolysosomal compartment [135]. Several studies have supported the idea that mtDNA is an endogenous agonist of TLR9 [136,137].

TLRs use different adapters. The most widely used is myeloid differentiation primary response protein 88 (MyD88). The binding between mtDNA and TLR9 triggers a signaling cascade, through MyD88, which culminates in the activation of mitogen-activated protein kinases (MAPKs) and nuclear transcription factor NF-κB [138,139].

The activation of the TLR9/NF-κB pathway promotes inflammation by inducing the expression of TNF-ɑ [140,141], IFN-I, and pro-inflammatory cytokines [142,143] (Figure 2).

TLR activation has been associated with immune responses that contribute to the attenuation of the pathological signs of AD [144]. In a series of studies, Scholtzova et al. investigated the possibility of using TLR9 as a therapeutic target in AD models, analyzing the effects of TLR9 activation in three different transgenic mouse models of AD [145,146,147]. Through monthly intraperitoneal injections of CpG oligodeoxynucleotides (CpG ODN), a TLR9 agonist, in three different mouse models of AD (Tg2576, 3xTg and Tg-SwDI mice), significant cognitive improvement and a reduction in fibrillar and soluble Aβ were noted. In contrast, microglial and macrophagic markers were essentially unchanged, indicating that no significant activation had occurred. In 3xTg mice, CpG ODN treatment also showed a reduction in the Tau pathology characteristic of this model [147].

Patel et al. confirmed the immunomodulatory role of the TLR9 CpG agonist ODN 2006 with experiments conducted on aged squirrel monkeys, an AD model with pathological characteristics quite similar to humans [148]. The administration of CpG ODN 2006 produced significant cognitive improvements, suggesting that this immunomodulatory approach may also have therapeutic potential in patients [148].

It should also be reported that, in AD models, some studies on the modulation of TLRs have produced side effects [149,150]. This can be explained by hypothesizing that, depending on the type of ligand used, the dose administered, the frequency of administration, and the stage of the disease, different signaling pathways can be activated. These pathways can alternatively go towards a neuroprotective inflammatory response with greater phagocytic activity and production of anti-inflammatory cytokines, or toward excessive inflammation and neurotoxicity.

## 5. Therapeutic Strategies

Several drugs are currently used in clinical trials for AD. Some of them target the two main pathological features of the disease, Aβ plaques and tau protein. Others turn towards anti-inflammatory mechanisms, while a considerable number is directed towards mitochondrial targeting.

At present, there are no treatments capable of stopping the progression of AD. The FDA has approved some drugs with different actions for clinical use. Tacrine, donepezil, carbalatine, and galantamine act as acetylcholinesterase (AChE) inhibitors, whereas memantine is an N-methyl-D-aspartate (NMDA) receptor antagonist. These drugs help to alleviate the symptoms of the disease, but cannot cure it. Lately, the focus has shifted to monoclonal antibodies targeting Aβ aggregation and, in 2021, the IgG1 monoclonal antibody aducanumab (BIIB037, ADU) was approved. Meanwhile, lecanemab (BAN2401) has completed a multicenter, double-blind Phase III study with 1795 participants showing a reduction in amyloid markers and cognitive improvement in patients with early AD, and a slightly better safety profile than aducanumab [151,152]. These findings convinced the FDA to authorize the use of the monoclonal antibody for patients with mild cognitive impairment (MCI) or mild dementia stage of AD.

Nonsteroidal anti-inflammatory drugs (NSAIDs) have shown a potential therapeutic effect in AD [153]. Epidemiological studies have suggested that long-term use of NSAIDs was related to a decreased risk of AD [14]. However, clinical studies have not confirmed such benefits, with the exception of indomethacin and naproxen [14,154]. Recently, a new, promising NSAID called itanapraced (CHF5074 or CSP-1103) has emerged. This drug has completed several Phase II clinical trials (NCT01303744, NCT01602393, NCT01421056), proving to be able to restore microglial function, increase phagocytosis, and decrease the production of pro-inflammatory cytokines, but no significant differences between treatment groups were found in neuropsychological tests [155,156].

Recently, pharmaceutical companies are looking with utmost care at the NLRP3 inflammasome, as a therapeutic strategy for several diseases, including AD. The aim is to inhibit the NLRP3 inflammasome and reduce the production of pro-inflammatory cytokines. In recent years, many NLRP3 inhibitors have been tested in preclinical studies; they have been discussed in depth by Barczuk et al. [97,104]. Currently, some NLRP3 inflammasome inhibitors are in Phase II trials for the treatment of AD [97,157].

### Drugs with Mitochondrial Action

As regards the molecules with mitochondrial targeting, in order to fulfil the purpose of this review, we will focus on antioxidant molecules and drugs acting on the permeability of the mitochondrial membrane, which can favor the release of mitochondrial DAMPs, as well as on drugs targeting the autophagic process that can regulate the inflammatory response driven by mitochondria.

Melatonin and its precursor N-Acetylserotonin (NAS) exert several potential anti-AD properties, including anti-oxidant capacity, and improve mitochondrial health by inhibiting mPTP. Moreover, melatonin has anti-inflammatory properties, suppresses NLRP3 activation, cytokine release, and shifts microglia towards an M2 anti-inflammatory phenotype [158]. Piromelatine, the extended-release melatonin, has shown a significant improvement in cognitive performance in patients with AD in a 24-week clinical trial [159]. However, a recently completed Phase II clinical trial (NCT02615002) showed no statistically significant progress in cognitive performance [160]. A long-term, prospective observational study to investigate the effects of melatonin on AD progression has just concluded, but results are not known yet (NCT04522960). A new randomized efficacy and safety study of piromelatine versus placebo, in participants with mild dementia due to AD is currently being carried out (NCT05267535).

Mitoquinone mesylate (MitoQ), based on coenzyme Q10 and the derivative of plastoquinone SkQ1, were shown to be effective antioxidants in vitro and in vivo, and are specifically targeted at mitochondria by covalent attachment to a lipophilic triphenylphosphonium cation [161,162]. 3xTg mice treated with MitoQ for 5 months showed reduced Aβ-induced cell death and oxidative stress in cortical neurons. Treatment with MitoQ also reduced Aβ accumulation, astrogliosis, and synaptic loss, leading to improved cognitive functions [163]. However, clinical trials have highlighted no significant progresses in cognitive decline so far (NCT00117403).

Astaxanthin, another mitochondria-permeable antioxidant, which can penetrate the BBB, also showed the ability to modulate neuroinflammation [164]. It is currently used in a randomized, double-blind, placebo-controlled trial to test its efficacy in AD (NCT05015374).

Hydralazine is an FDA-approved drug with neuroprotective effects derived from different actions. Hydralazine is a strong antioxidant, improves mitochondrial health, and activates autophagy decreasing intracellular aggregate [165]. A Phase III, triple-blind, parallel double-armed randomized clinical trial is currently underway (NCT04842552).

Glutathione (GSH), an endogenous antioxidant, is fundamental for mitochondrial function, and its deficiency is linked to AD [166]. N-acetyl-cysteine (NAC) is a compound that can cross the BBB and provides precursors for GSH synthesis. Several studies have shown that NAC shows antioxidant and anti-inflammatory activities, protects from Aβ-induced toxicity, reduces Aβ levels, decreases the amount of phosphorylated tau, and preserves cognitive function [167,168,169]. A randomized, double-blind study has evaluated a combined therapy of a nutraceutical formulation composed of NAC, folate, vitamin E, vitamin B12, s-adenosyl methionine, and acetyl-L-carnitine in AD subjects. Subjects who received this formulation showed improvements on their dementia rating scale [170]. There is currently an ongoing randomized clinical trial to evaluate the effects of NAC supplementation for 24 weeks compared to placebo in patients with AD. This study will measure the changes in cognitive function, metabolic and mitochondrial activity, oxidative stress, and brain inflammation (NCT04740580).

Nicotinamide adenine dinucleotide (NAD+), a cofactor for several proteins including sirtuins, is directly involved in mitochondrial biogenesis and mitophagy [171]. The NAD^+^ precursors, nicotinamide riboside (NR) and nicotinamide mononucleotide (NMN), are powerful inducers of mitophagy. In APP/PS1 mice, NR improved cognitive functions by reducing cortical Aβ deposits and increasing mRNA levels of the mitophagy proteins PINK1 and LC3 [172]. Moreover, NR treatment reduced expression of pro-inflammatory cytokines, and decreased activation of microglia and astrocytes. NR treatment also reduced NLRP3 activity and cGAS-STING activation [79]. Similarly, NMN reduced inflammation and Aβ accumulation in the brain, inhibited neuronal apoptosis, preserved mitochondrial function, and improved cognitive impairment in AD mice [173,174]. There is currently an ongoing Phase I clinical trial to evaluate the effect of NR on brain energy metabolism, oxidative stress, and cognitive function in individuals with MCI and mild AD (NCT04430517). Moreover, a Phase I/II clinical trial is underway to evaluate if the microcrystalline form of NMN (MIB-626) penetrates the BBB and estimate the effect on circulating biomarkers of aging (NCT05040321).

Several studies suggest that some hypoglycemic drugs can improve mitochondrial performance by preventing both the ROS production and mitochondrial dysfunction [54,175,176,177]. Moreover, different clinical trials have shown that administration of intranasal insulin, metformin, or thiazolidinediones (pioglitazone, and rosiglitazone) in patients with MCI and AD can improve cognition performance and memory [178]. The action of insulin on mitochondria has been widely discussed in a precedent review [54].

Metformin stimulates autophagy by acting on AMP-activated kinase (AMPK), improves mitochondrial function, and reduces inflammation [179,180]. However, two Phase II clinical trials (NCT01965756 e NCT00620191) showed no significant improvement in cognitive function after metformin administration [181,182], but highlighted some side effects (gastrointestinal symptoms and vitamin B12 deficiency) [183,184]. To date, there are three ongoing clinical trials, currently in the early stages, investigating the effects of metformin on cognitive function and brain health in individuals with MCI or at-risk for AD (NCT04511416, NCT05109169 and NCT04098666).

In Table 1, we summarized the current therapeutic approaches targeting mitochondria in treatment of AD, described in the present chapter.

To sum up, several molecules are able to improve mitochondrial function, showing positive effects in preclinical models of AD. There are many limitations to overcome, such as low bioavailability, rapid metabolism, and poor ability to cross the BBB. While waiting to develop new formulations aimed at overcoming these limits, adopting a healthy lifestyle (Mediterranean diet, caloric restriction, and physical exercise) can help preserve cognitive function.

## 6. Conclusions

Mitochondrial dysfunction and neuroinflammation are two important factors in neurodegenerative diseases such as AD. In the scientific field, there is still debate about the triggering event. Glia recognize mitochondrial DAMPs, including mtDNA, released from damaged mitochondria, activating various inflammatory pathways through PRRs.

The dichotomous nature of the cells implicated in inflammation suggests that intervening in the inflammatory process by suppressing the activity of glial cells is not an easy and assured strategy for success. This could explain the partial failure of clinical trials of anti-inflammatory drugs in AD patients [185,186,187,188]. Intervening in the most appropriate time window, during the pathological process, requires a better understanding of the timing and succession of the activation mechanisms in the development of the disease. Acting in the initial stages of the pathology can help to distinguish the neuroprotective or neurotoxic microglial phenotype. To stimulate the first and block the second, through selective interventions, could be a winning strategy against neurodegeneration. Furthermore, understanding how to modulate the levels of mtDNA and inflammatory DAMPs could also open the way to new intervention possibilities. For example, in recent work, Zheng et al. used small molecules capable of intercalating into mtDNA to stimulate the release of mtDNA fragments, activating the cGAS-STING pathway and modulating a specific immune response [189].

Further studies are needed to better understand these processes and investigate the interaction pathways among these mechanisms. This could allow us to identify key molecules that could become therapeutic targets for the treatment of neurodegenerative diseases and help us find the most effective strategies to tackle the great challenge of AD.

## Figures and Tables

**Table 1:** Summary of current clinical trials involving mitochondria-target therapies for AD treatment.

Drug	Activity	Patients	ClinicalTrials.gov ID
Melatonin	Antioxidant; mPTP inhibitor.	60 partecipants Age: 18 years and older with AD dementia or MCI	NCT04522960
Piromelatine		225 participants; Age: 60–85 years with cognitive decline	NCT05267535 Phase II/III
Coenzyme Q10	Antioxidant	100 participants; Age: 50–80 years with MCI or AD	NCT06040905
Astaxanthin	Antioxidant; mPTP inhibitor.	50 participants; Age: 60–90 years with dementia	NCT05015374
Hydralazine	Activates autophagy; Regulates antioxidant production by NRF2	424 participants; Age: 49 Years and older with diagnosis of AD	NCT04842552 Phase III
Glutathione (GlyNAC)	Antioxidant	52 participants; Age: 55–85 years with progressive memory loss	NCT04740580 Early Phase I
Nicotinamide mononucleotide (MIB-626)	Induces mitophagy; Preserves mitochondrial function	50 participants; Age: 55–85 years with AD	NCT05040321 Phase I/II
Nicotinamide Riboside	Boosts NAD^+^ levels; Activates mitophagy; Regulates mitochondrial function	50 participants; Age: 55–89 years with MCI or Mild AD	NCT04430517 Early Phase I
		80 participants; Age: 50–85 years with AD	NCT05617508
Insulin	Regulates mitochondrial function	40 participants; Age: 55–85 years with mild MCI or Mild AD	NCT05006599 Phase II
Metformin	Antioxidant; Preserves mitochondrial morphology; Induces mitophagy.	242 participants; Age: 60–80 Years with MCI	NCT04511416 Phase III
		600 participants; Age: 60–79 Years with risk factors for dementia	NCT05109169 Phase II
		326 participants; Age: 55–90 years with MCI	NCT04098666 Phase II/III

Abbreviations: Mitochondrial permeability transition pore (mPTP); Mild cognitive impairment (MCI); NF-E2 p45-related factor 2 (NRF2); Nicotinamide adenine dinucleotide (NAD^+^); Alzheimer’s disease (AD).

## Data Availability

Not applicable.
